# Storied Bodies and Temporal Lives: A Narrative Inquiry Exploring the Experiences of Young Adults Living with an Eating Disorder

**DOI:** 10.1177/23333936261419040

**Published:** 2026-02-18

**Authors:** Sydney Gaudet

**Affiliations:** 1 University of Prince Edward Island, Charlottetown, PE, Canada

**Keywords:** eating disorder, young adult, narrative inquiry, embodiment, temporality, Canada

## Abstract

An eating disorder is a type of mental illness, characterized by a disturbance in eating related behaviour, resulting in significant physical and psychological impairments. Despite this recognition, eating disorders remain poorly understood and plagued by high mortality and low recovery rates. Furthermore, individuals living with an eating disorder rarely have opportunities to shape study design or inform treatment. This study used narrative inquiry to explore the experiences of two young adults, aged 18 to 30, living with Anorexia or Bulimia Nervosa, and self-identified as living with an eating disorder. Participants were interviewed five times over 3 months, and narrative accounts were co-composed through a collaborative process emphasizing relational engagement and therapeutic alliance. By centring the lived experience of participants this study engages with the complexity of living with an eating disorder rather than taking it for granted. Resonant narrative threads that were constructed through co-creation of knowledge are discussed. This article extends traditional research approaches by responding to participants’ priorities, fostering co-constructed understanding, and offering pathways to inform research and care design that reflect the nuanced realities of living with an eating disorder.

## Background

. . . it wasn’t like I wanted to die from my eating disorder, but I also knew that I was damaging my body a lot and that that might happen and not being opposed . . . It’s like you’re assuming the risk pursuing you’re eating disorder. . . like you’re signing a waiver saying I’m going to participate in this and if I die it’s fine. . . (Emma, young adult living with an eating disorder)

As I recall the words spoken by Emma, I remember not only listening to her words but also observing the way her body conveyed them. She discussed death and dying with an almost startling indifference, stating it as plainly as someone might remark, ‘the sky is blue’ on a cloudless afternoon. Her metaphor of ‘signing a waiver’ of risk was familiar, echoing countless conversations I’ve had as a psychiatric and mental health nurse with patients who wrestle with their own ambivalence toward treatment, or, perhaps more accurately, with the intricate dynamics of their relationship with their eating disorder. Healthcare professionals often label this ambivalence as denial, a term that flattens its complexity. But Emma’s account makes it clear: what some call denial is not a simple refusal to see the truth. It is something far more layered.

Eating disorders are ‘complex’ mental illnesses characterized by persistent disturbances in eating or eating-related behaviour, and often begin in adolescence (American Psychiatric Association [APA], 2022; Klein et al., 2021; Medway et al., 2019; Sternheim et al., 2021). Mortality rates are among the highest of all mental illnesses (Couturier et al., 2020; Obeid et al., 2020; Sternheim et al., 2021), with early detection and treatment key to improving prognosis (Canadian Eating Disorders Alliance [CEDA], 2019; Couturier et al., 2020; Klein et al., 2021; Malson et al., 2022). Treatment options for those diagnosed with an eating disorder are varied and limited and have high rates of treatment dropout and relapse (Klein et al., 2021; Malson et al., 2022; Sternheim et al., 2021; van Doornik et al., 2021). Despite the seriousness and prevalence of eating disorders, understanding continues to remain limited about the nature of eating disorder illness and what causes development (Couturier et al., 2020; de Vos et al., 2017; van Doornik et al., 2021).

Research shows that both the public and healthcare professionals hold misconceptions about eating disorders, often viewing them as voluntary and attributing responsibility to the individual, leading to the assumption that treatment should be easy (Brelet et al., 2021; Dimitropoulos et al., 2016; LeBlanc, 2014). Preconceived ideas about who is living with an eating disorder contribute significantly to stereotypes and stigma and often predict adverse outcomes and a poorer prognosis (Brelet et al., 2021; LeBlanc, 2014). Furthermore, eating disorder research often conflates sex and gender (Burnette et al., 2022; Urban et al., 2024). Meanwhile, eating disorders can manifest among diverse individuals with diverse backgrounds and body types (Hartman-Munick et al., 2021; LeBlanc, 2014).

Treatment is often based on a western medical model (Fuchs, 2022), with diagnostic tools, screening tools, and eating disorder conceptualization largely developed and evaluated from cisgender female patients. As a result, those who identify as men or boys, members of ethnic and visible minorities as well as members of 2SLGBTQ+ community are all underrepresented in eating disorder literature and research (Arnow et al., 2017; Corcoran et al., 2021; Darcy et al., 2012; Griffiths & Yager, 2019; Hartman-Munick et al., 2021; Joy et al., 2021; LeBlanc, 2014).

Qualitative research on eating disorders emphasizes the importance of including lived experience in both research and care (Obeid et al., 2020; Ortiz et al., 2019). Yet, those with lived experience rarely have the opportunity to shape research, inform treatment approaches, or identify key priorities (CEDA, 2019; Obeid et al., 2020; Ortiz et al., 2019).

Individuals living with an eating disorder discuss an intense desire to participate in research that promotes the opportunity to obtain accurate and holistic data nuanced with an understanding of lived experience (Ortiz et al., 2019). Building a therapeutic relationship or therapeutic alliance is necessary for participants to feel safe about sharing their lived experience (Koruth et al., 2012; Ortiz et al., 2019). Furthermore, individuals express extreme deterrence to participating in research if they feel a lack of validation from researchers or are perceived as a form of data instead of an individual with lived experience (Ortiz et al., 2019).

Despite frequent descriptions of eating disorders as ‘complex,’ the term often becomes a catch-all phrase, inadvertently avoiding engagement with the messy realities of lived experience. Law and Mol (2002) propose that complexity should be understood not as an epistemological concern (how accurately reality is represented) but as an ontological one, meaning that reality is multiple and enacted through relations. In the context of eating disorders, this means understanding them not as fixed entities but as emergent through relational, social, and material processes (Lester, 2021). Such an approach calls for a shift from epistemological certainty toward ontological openness, one that remains largely absent from current eating disorder literature.

Narrative inquiry as defined by Clandinin and Connelly (2000) offers a way to engage with, rather than reduce, the complexity of lived experience. It is a relational methodology oriented towards intimacy, trust, and collaboration, recognizing that these qualities are not inherent outcomes but potential orientations that can develop through relational engagement. The research relationship evolves as the researcher and participant come together in a non-hierarchal manner over time. This relational aspect creates a deep connection between the researcher and participant, and as a result, a research relationship is developed where personal experiences and memories can be described in great emotional detail (Clandinin, 2013; Clandinin & Connelly, 2000). Instead of approaching narrative as a tool to reflect or reinterpret experience, narrative inquiry enacts a way of being, one that understands stories as generative, both shaping and giving meaning to experience. Stories are not fixed representations of experience; they are the very means through which experience is shaped. Thinking narratively involves attending to how people live, tell, retell, and relive their stories in context (Clandinin, 2013; Clandinin & Connelly, 2000). This focus shifts us from asking what an experience means to asking how it is made and remade in relation.

Rather than seeking generalizability, narrative inquiry aims to illuminate poorly understood phenomena through deep, storied accounts situated within temporal, social, and environmental contexts. Although narrative inquiry has the potential to enhance understanding and capture the ‘complex’ nature of eating disorders, care, and recovery, few researchers have used Clandinin and Connelly’s (2000) specific form of narrative inquiry in eating disorder research.

For nursing, this methodological shift is particularly significant. Nurses hold a central role in eating disorder care, spending a significant amount of time with patients in relation to other healthcare professionals, navigating tensions between biomedical protocols and recovery-oriented practice (Corral-Liria et al., 2021; Jennings, 2017; Ryan et al., 2006; Wu & Chen, 2021). Yet research exploring nursing care in this context remains limited, with nurses reporting challenges, exhaustion, and moral distress when balancing these competing demands (Davén et al., 2022; Gustafsson et al., 2021; King & Turner, 2000). Narrative inquiry enables nurse researchers to engage relationally, co-constructing knowledge that attends to the lived experiences of both patients and nurses. By centring these storied experiences, nursing research can deepen understanding, expand awareness, and generate insights that resonate with practice, ultimately shaping care in ways that reflect the realities of those living with eating disorders.

Narrative inquiry is grounded in the understanding that humans ‘lead storied lives’ (Clandinin, 2013, p.13). In this sense, ‘story’ becomes ‘a portal through which a person enters the world and by which their experience of the world is interpreted and made personally meaningful’ (Clandinin, 2013, p. 13). As Okri (1997) offers, ‘we live by stories’ and ‘we also live in them’ (p. 46).

In this paper, I detail a narrative inquiry study with two young adults living with an eating disorder, Julia and Emma. I present resonant narrative threads that emerged through our collaborative work and reflect on how creating relational spaces allowed for deep sharing of experience. This positioning informs not only the content of this article but also its design. By integrating reflexivity throughout, I aim to extend more traditional aspects of research reporting and illustrate what narrative inquiry can offer to eating disorder research and nursing practice.

## Methodology

Philosopher Dewey’s (1938) theory of experience provides the philosophical and ontological underpinnings to narrative inquiry, specifically, his two criteria of experience: interaction and continuity enacted in situations. Interaction refers to the understanding that while people are individuals, they cannot be understood in isolation, they are always in a social context. Continuity refers to the relational, temporal, and continuous flow of experience (i.e., experiences grow out of other experiences, leading to further experiences). As such, a narrative ontology implies that experiences are continuously interactive, with the narrative inquiry researcher focusing on the way the relational, temporal, and continuous features of a Deweyan view of experience are manifested as narrative form (Caine et al., 2013; Clandinin, 2006; Clandinin & Murphy, 2009). Clandinin and Connelly (2000) developed a three dimensional metaphorical inquiry space to conceptualize narrative inquiry. Narrative inquirers position themselves within this three dimensional inquiry space of interaction (the personal and social), continuity (past, present, and future), and place (situation) to inquire into both the researcher and participants storied life experiences, and understand each story told and lived within larger social, cultural and institutional narratives (Caine et al., 2013; Clandinin, 2006; Clandinin & Caine, 2008; Clandinin & Connelly, 2000; Clandinin & Murphy, 2009; Clandinin, et al., 2016, 2017; Connelly & Clandinin, 1990).

Embedded within Dewey’s theory of experience is acknowledgment of embodiment. Dewey’s view of experience recognizes the embodiment of the person in the world, meaning how experience is shaped through the engagement of the senses. Dewey suggests that while our senses (i.e., sight, smell, taste) are physical and physiological mechanisms, they are also tools in which we filter, add to, and refract stories in different ways (Caine et al., 2013; Clandinin, 2006; Connelly & Clandinin, 1990; Dewey, 1934). Therefore, narrative inquiry researchers attend to how embodied experiences shape the life stories of both the researcher and participants (Caine et al., 2013; Clandinin, 2006; Clandinin et al., 2017).

### Recruitment

To align with the aims of narrative inquiry, a small sample size was intentionally chosen. Narrative inquiry prioritizes depth over breadth, seeking to generate rich, storied accounts that can open up understandings of phenomena rather than generalized findings (Clandinin & Connelly, 2000). Because the methodology requires sustained engagement, often across multiple conversations, reflections, and negotiated texts, a smaller number of participants ensures that the researcher can attend closely to the evolving relational and contextual dimensions of experience. This level of engagement allows for the co-construction of narratives that honour complexity and nuance, which would be difficult to achieve with a larger sample. Guided by these principles, two young adults living with an eating disorder and residing in Prince Edward Island were recruited to participate in this study.

After receiving ethical approval from the University of Prince Edward Island (UPEI) (FILE #: 6011602) in June 2022, participant recruitment began. A recruitment poster advertising this study was developed and shared with the Director for Community Mental Health and Addictions, who disseminated this poster to mental health therapists working across the province. The poster was also displayed in multiple locations frequented by young adults, such as community gyms, coffee shops, grocery stores, pharmacies, and post-secondary institutions.

After 3 months without successful recruitment, an amendment was submitted to the Research Ethics Board on September 2, 2022, requesting permission to share the recruitment poster via social media (Facebook, Twitter, and Instagram) and through an Eating Disorders Anonymous group. Approval was granted on September 7, 2022. Shortly thereafter, two individuals contacted me via email to express interest in participating. Following email correspondence, phone calls were scheduled to provide additional details about the study.

During these phone conversations, I outlined the research design, purpose, and expectations and reviewed the inclusion criteria. These criteria required participants to be: aged 18 to 30 years, able to read and speak English, have a diagnosis of either Anorexia Nervosa or Bulimia Nervosa as defined by the *Diagnostic and Statistical Manual of Mental Disorders* (5th ed.) Text Revision (DSM-5-TR) (APA, 2022), self-identified as living with an eating disorder, and could not be in active treatment nor have received active treatment within the last 6 months. Active treatment was defined as inpatient hospitalization to either a medical or psychiatric unit for refeeding, medical stabilization, or psychiatric stabilization.

Both individuals met these criteria, reviewed the letter of information, and provided informed consent. Participant interviews were scheduled shortly thereafter. The recruitment strategy and emphasis on early scheduling were informed by my prior clinical experience as a registered psychiatric and mental health nurse and supported by scholarly literature identifying challenges in the recruitment and retention of individuals living with eating disorders. These challenges were later confirmed by participants, who shared that they had read the recruitment poster prior to the original amendment multiple times before reaching out for additional information.

Initially, I assumed that participants would prefer pseudonyms to maintain anonymity. However, one participant had chosen to keep their true name, explaining that this decision was a step in establishing control, separating their identity from their eating disorder, and helping them not to feel ashamed of their lived experience. As this choice was discussed, I spoke about what is referred to as ‘blurring’ in Clandinin and Connelly’s (2000) form of narrative inquiry. To help preserve anonymity while keeping the participant’s name, the I blurred times, places, and identities (Clandinin, 2013; Clandinin & Connelly, 2000). When I came together with both participants to review their final narrative account and research texts, each participant confirmed their comfort with how I had presented the telling of their storied life experience, inclusive of the blurring strategies employed throughout. Further, throughout the narrative inquiry study, I did not identify which participant had chosen to keep their true name.

### Data Collection and Field Work

*Field texts* in narrative inquiry are akin to data collection in other research methodologies (Clandinin & Connelly, 2000). Field texts are composed from within the three dimensional inquiry space attending to temporality, sociality, and place (Caine et al., 2013; Clandinin, 2006; Clandinin & Caine, 2008; Clandinin & Connelly, 2000; Clandinin et al., 2016). As a reflexive methodology, narrative inquiry is recursive in the movement between field and field text throughout the entirety of the inquiry. The narrative inquirer utilizes the three dimensional inquiry space as an anchor in navigating the tensions of moving from field to field text, interim and final research texts, and making sense of *resonant threads* as they emerge. There is a myriad of what constitutes field texts in narrative inquiry (Caine et al., 2013; Clandinin, 2006; Clandinin & Caine, 2008; Clandinin & Connelly, 2000; Clandinin et al., 2016). In this narrative inquiry, field texts were composed of transcripts of conversational interviews and a reflexive journal.

### Data Analysis and Interpretation

Composing research texts is the interpretive-analytic consideration in narrative inquiry more commonly referred to as data analysis in other research methodologies. Moving from field texts to research texts is a complex and reflexive process with no single defined approach. Instead, the narrative inquiry researcher returns to field texts repeatedly throughout the narrative inquiry study and shares these interpretations with participants (Clandinin & Connelly, 2000; Clandinin et al., 2016). Research texts are negotiated with participants, who are the most influential voice in moving to the final research texts (Clandinin, 2013; Clandinin & Connelly, 2000).

Response communities are a way to engage in responsive and responsible dialogue about interim research texts. These sustained discussions help the beginning narrative inquiry researcher recognize and shape their final research texts (Clandinin & Caine, 2008; Clandinin & Connelly, 2000). For this inquiry, my Master of Nursing thesis supervisor Dr. Christina Murray PhD, RN served as my response community. Additionally, I met with each participant individually after composing what is referred to as a *narrative account* of the co-constructed experiences between the researcher and participants during their time together. This was done by sharing unfinished texts with participants, and they provided input and feedback on the interpretation of the transcripts and writing of the final research text (Clandinin, 2013).

## Meeting Julia and Emma

### Entering into a Research Relationship with Julia

It’s a beautiful fall day outside in late September of 2022. It’s utter chaos in my house, the effects of having two young toddlers running amok. My phone rings. I do not recognize the number, and consider directing the call to voicemail, instead I answer the phone. The voice on the other end of the line is quiet, shaky, shy and holds an air of vulnerability, expressing interest in a research study about eating disorders that they had seen in a poster. I felt myself instinctively shift into making my mind and body present in the moment, an embodied response to the characteristics of the voice I was hearing on the other end of the telephone line. Years of working as a psychiatric and mental health nurse had trained me to recognize this, a person stepping, however briefly, outside the grip of their eating disorder.

I remember thinking, *be careful*, and quietly slipping into a corner of my house where I could shut the door and give my full attention to this stranger.

I gently asked their name. A small, embarrassed laugh followed.

Julia: ‘Oh, it’s Julia.’

Sydney: ‘Hi, Julia,’ I replied. ‘It’s nice to hear from you. My name is Sydney.’

This first conversation laid the foundation for what would become Julia’s co-participation in this research study. Over the next 3 months, we met five times, each conversation stretching between 50 and 90 min.

### Entering into a Research Relationship with Emma

Emma’s initial contact came through email, expressing interest in the study. We scheduled a phone call, and as we spoke, I noticed a similar automatic response in me to her tone and cadence, much like when I first spoke with Julia. Yet, Emma carried a different presence.

Unlike Julia, Emma was well-versed in research, steering our conversation with pointed questions to clarify aspects of the study. She spoke openly about her relationship with her eating disorder, her words marked by a clarity and confidence that made me curious about how her experiences contrasted with Julia’s. Emma and I met five times over a 3 month period, with each conversation lasting between 50 and 90 min.

At the time of the study, both Julia and Emma were post-secondary students. They recalled the early stirrings of their eating disorders in childhood and adolescence, with their struggles becoming more entrenched in young adulthood. Their experiences were shaped by a web of socio-cultural and economic pressures, as well as early family attachment dynamics. Both were diagnosed with Anorexia Nervosa, a common type of eating disorder, yet their journeys to that diagnosis, and their experiences of care, were strikingly different.

Within this narrative inquiry, co-participants discussed primary motivation for participation was due to their hope that sharing their storied experiences could help others and attend to deeply entrenched stereotypes and stigma about eating disorders, anorexia particularly.

Such is exemplified by Emma, who discussed the importance of representation, and the experience of having an eating disorder when you are of a larger body shape and weight.

Emma: Without having it (representation), it leaves so many people without any help that need help. Even if you’re not underweight, your eating disorder can destroy your body, and it can destroy your organs and all that stuff. I think it’s important too because I think there’s so many more people that have eating disorders that don’t feel validated in their experiences. Like you don’t feel good enough, for an eating disorder right if you don’t see these other stories. So, I feel like the lack of representation just pushes people further into isolation and into their eating disorders. If there was a way for people to get help earlier or see people like me, they might reach out for help earlier

#### Constructing Narrative Beginnings

One of the central tenets of narrative inquiry is that researchers enter ‘in the midst’ (Clandinin & Connelly, 2000, p. 63) of the storied lives of participants and, as such, are co-participants in the research process. Entering these relational spaces comes with a responsibility to ethical considerations and is continually guided by ethics and attitudes of openness, mutual vulnerability, reciprocity, and care (Clandinin & Caine, 2013; Finlay & Dela Cruz, 2023). The writing of these autobiographical narratives are known as the composing of *narrative beginnings*, a deeply personal and, at times, challenging undertaking.

For me, these tensions revolved around my personal and social experiences, my family life, my peer relationships, and my work as a psychiatric and mental health nurse. Furthermore, the emotional complexity of caring for individuals with eating disorders throughout the lifespan, which can be gathered from the following excerpt:

Sydney: . . .Through constructing my narrative beginnings, and throughout this narrative inquiry, I have come to recognize the profound impact that my early experiences with peers who had eating disorders had on my life. It wasn’t until I began this study that I fully understood how these experiences had shaped my career and my deep commitment to working with individuals living with eating disorders. . .

Engaging in relational research also brought forth challenges as I navigated the boundaries between professional practice and relational inquiry. Having been conditioned through professional norms and hegemonic research traditions to maintain firm distinctions between the personal and professional, I found myself grappling with the complexities of relational engagement. This struggle is captured following a conversational interview that I had with Julia:

Sydney: I found myself to be emotionally affected through the intimacy, emotional vulnerability, and deep connection that occurred following such a brief time together. I situated myself within the narrative inquiry three dimensional space and looked inward regarding my personal experiences and the experiences of a narrative inquiry researcher. The first question that came forward to me during this reflective period was simple; why was this impacting me? As a registered psychiatric and mental health nurse, I have heard stories of adversity and resiliency frequently. So, what about this moment was different? What I came to realize was that I was navigating the tensions of what it means to perform relational research. As a nurse, my boundaries when engaging in my professional work are clearly defined and parameters established. During this study, I was engaging in the relational dimensions of narrative inquiry, attending to my experiences as the narrative inquiry researcher. . .

Through constructing my narrative beginnings, I deepened my awareness of my positionality in relation to the phenomena of interest within this study (Clandinin & Connelly, 2000). I am a white, cisgender woman, born and raised in Canada. I recognize that my positionality is shaped by privilege and access to resources, and I strive to remain critically aware of my own biases and assumptions, knowing that they inevitably shape the lens through which I approached this research.

## Findings

As I read and re-read the field texts, both individually and collectively, I engaged in an iterative process situated within the three-dimensional narrative inquiry space of interaction, continuity, and situation (Clandinin & Connelly, 2000). Through this process, I began narratively coding the stories, attending to themes, patterns, and outlying concepts that emerged as participants and I co-constructed understandings of their experiences. These codes were not predetermined but developed as new insights surfaced through our ongoing engagement. While each storied experience remained unique, I also attended to resonances between Julia and Emma’s narratives, retaining those shared threads. As I formulated final research texts, I was particularly drawn to two interrelated narrative threads that resonated strongly across Julia and Emma’s shared experiences, temporality and embodiment.

### Temporality of Eating Disorder Illness

One of the most prominent revelations illuminated to me was how each participant lived within non-linear forms of temporality while sharing their storied experiences. During conversational interviews, participants moved with overlapping fluidity between past, present, and sometimes future tenses while sharing. At times, this movement felt seamless and, at other times, messy. Sometimes, the speed along this temporal flow was measured, and others with a veracity of pace the I could only sit and listen. All the while, I felt this innate urge to gather the overlapping folds of time, pull them apart, and then place them back together, almost like a puzzle, in a rational, linear telling.

At the time, the I couldn’t identify that such a need reflected the hegemonic ideas of time instilled in me through traditional research methods and dominant biomedical regimes where eating disorder conceptualization and care are bound within curative, disease-oriented frameworks. Within these frameworks, eating disorder conceptualization and recovery are tied to diagnosis and cure. However, to Julia and Emma, existing *within* this complexity of time seems like a much more realistic and nuanced representation of eating disorder experience and conceptualization of eating disorder recovery. Rather than striving for a life entirely without an eating disorder, they acknowledged a persistent, albeit shifting, relationship with it

This finding resonates with emerging qualitative literature that explores temporality in eating disorder recovery, challenging the notion of recovery as a fixed or linear state (Eli & Lavis, 2022; Lavis, 2018; LaMarre & Rice, 2016). Much of the research examining temporality in eating disorders has been situated within longitudinal studies focusing on recovery trajectories. Within this literature, recovery is recognized as an elusive concept, with significant debate regarding what defines eating disorder recovery (de Vos et al., 2017; Eli & Lavis, 2022; Elwyn et al., 2024; LaMarre & Rice, 2016). Traditionally, recovery has been conceptualized through a medical model, prioritizing symptom remission and weight restoration (de Vos et al., 2017; Kenny et al., 2019; Sternheim et al., 2021). Yet qualitative studies have demonstrated that defining recovery solely around symptom management is insufficient (de Vos et al., 2017; Kenny et al., 2019; LaMarre & Rice, 2016). In research where voices of those with lived experience shaped understandings of recovery, discrepancies emerged between clinically assessed and subjective recovery (Bardone-Cone, 2012; Gillespie & Robinson, 2022).

The experiences shared by Julia and Emma extend this conversation by foregrounding ‘ongoingness’ as central to how they conceptualize recovery. When reflecting on what recovery meant to them, neither participant defined it by a target weight or the absence of eating disorder symptoms. Instead, they envisioned recovery as learning to live with the presence of an eating disorder in ways that allowed autonomy and agency over its characteristics. This perspective aligns with and expands upon existing scholarship that critiques binary notions of illness and recovery (Eli & Lavis, 2022; LaMarre & Rice, 2016) and calls for frameworks that attend to multiplicity, non-linearity, and lived temporalities.

Julia: . . .Food’s always going to be something in my life, so you know I feel there’s going to be stages where like it is better, but I don’t feel like it is ever fully going to go away. . . I don’t think I’ll ever be cured; I think I’m just going to have to find ways to manage to live with it. . . ‘Cause you know, recovery isn’t just going to go, you know (gesturing a straight line up) . . .

Emma: . . . Maybe I can make it less of my life going forward but it’s still, it’s just automatic like thoughts and that kind of stuff. I feel it’s always going to be there. I think there’s always going to be triggers you know . . .

Emma likened her idea of recovery to that of a car accident, stating

Emma: You know like someone who’s recovered from a car accident or something they’re still probably going to have some injuries and stuff that they’re going to deal with. Their pain or whatever injuries, but you know they’re stable, they’re able to function again. I think with an eating disorder there’s always going to be that little bit that’s still there and you’re probably going to have flare ups, but I think that’s because there’s not really a way to erase my history, you know I’ll always have the memory of it . . .

While working through the emerging resonant threads of temporality and navigating the recursive, temporal waves of Julia and Emma’s storytelling experience, I began questioning my own ideas about eating disorder conceptualization, treatment, and recovery.

Sydney: ‘How do we define eating disorder recovery? Who should define eating disorder recovery? Is it possible to ever recover from an eating disorder illness when recovery is defined as the absence of?’

### Embodiment

Within eating disorder literature, there is growing attention to embodiment, understood as how individuals experience their bodies and make sense of the world through the body’s active, meaningful engagement with it (Fuchs, 2022; Osler, 2021). Scholars within eating disorder literature have highlighted that for many living with an eating disorder, embodiment is marked by disruption or alienation, where bodily signals are muted or overridden, and appearance becomes a primary measure of self-worth (Eli, 2015; Katznelson et al., 2021). These dynamics were vividly present in Julia and Emma’s stories. Both described a relationship with their bodies that extended far beyond dissatisfaction; it was characterized by a profound sense of shame and intolerance toward simply existing in their bodies.

This experience aligns with research noting how eating disorders often involve an attempt to regulate or reshape bodily experience through practices such as calorie counting, food restriction, purging, excessive exercise, or substance use (Gattario et al., 2020; Rossi et al., 2021). For Julia and Emma, these practices were not only about altering appearance but about controlling or silencing embodied sensations, a theme echoed in studies that discuss intentional suppression of hunger cues and other bodily signals as a strategy for coping with perceived loss of control (Eshkevari et al., 2014; Malecki et al., 2018). Similarly, participant narratives reflect what Osler (2021) describes as the objectification of the body, a project to be mastered rather than a lived, sensing subject.

While similarities can be drawn from Julia and Emma’s experience, each experienced care organization and treatment differently based on their different body shapes and weight. For Emma, having a body shape and weight outside the stereotypical image of the body shape and weight of someone who experiences anorexia nervosa was a dominant narrative thread in her storied experience.

Emma: I was overweight before it started, all the restrictions and stuff. Not super overweight but still overweight and so you know I lost that 50 to 60 pounds which then brought me down to kind of the between underweight and normal weight . . . and so no one really noticed for a long time. I mostly got congratulations, which only then just encouraged everything. . . people were more like ‘oh cool weight loss journey, we love that for you. . .

Emma continued: ‘. . . it was really hard to access any help, I had to ask for it directly. I wasn’t at a dangerously low weight, but no one really considered all of the damage I was still doing to my body. . . I was still getting all of those effects from it. . . I was bruising really easily, numbness, fainting, hair loss, all that stuff . . .’

Emma described measuring the success of her eating disorder through the protrusion of specific body parts: collarbones, ribs, and hips.

Emma: I covered up pretty good. I have always had broad shoulders and things like that so I felt if I wore bigger clothes, I could look bigger. It helped to show just how small I was under my clothes, my ribs were out, my collarbones were super prominent, I had not quite a 6 pack, but I had visible abbs that generally people don’t have. . .

For Julia, her experience of body dissatisfaction has existed as long as she can remember and is entangled within the intersections of embodiment, gender, and sexuality:

Julia: I kind of repressed my sexuality for a really long time. I did actually know that I was gay a really long time ago like eight or something. I didn’t know it was a thing, but I knew that I had like crushes on girls in my classes and not guys . . . I wanted to you know maximize my femininity, I don’t know why (she paused), I cannot, I can’t explain it now, but it was just me trying to repress it . . . I didn’t feel comfortable with myself at that time and that just caused me to get upset when people were interested in me because I didn’t really feel like myself you know? I don’t know if that make sense, but I never felt like comfortable with my body or the way I looked so I didn’t know how other people could. . .

Julia’s narrative illustrates how gendered ideals of femininity operated as a regulatory force in her efforts to ‘maximize’ certain features of her appearance, even as she worked to suppress her sexual identity. As Burnette et al. (2022) and Urban et al. (2024) note, body dissatisfaction is not only linked to internalized beauty norms but also to broader gendered social dynamics that compel individuals to align with heteronormative femininity. Julia’s discomfort when others expressed interest in her reflects this tension; her body became a site where gendered expectations, heteronormativity, and sexuality collided, shaping both her sense of self and her embodied experience.

During one conversational interview, I experienced a visual representation of my burgeoning ideas of how embodiment and temporality can be complexly entangled. Julia was describing in great intensity her recollection of wanting to physically cut off skin tissue to reach an idealized body shape as a child.

Julia: I’ve always just been concerned about my body and the way I looked, my weight, ever since I was a kid you know? I remember in elementary school, I would look at how other girls looked in my class and I would think, I don’t look like that, or I wish I looked like that. . . I would just pick and choose from other people. I would look at my legs and think that I had too much fat on my legs, or my arms, above my shoulders and stuff. I wanted a flat stomach when I was, you know 10, just a little kid who shouldn’t really care about that. But I did. I would touch my stomach and be like OMG!. . . I would grab my stomach and grab a pair of scissors and think ‘what if I just cut this off?!. . .

As Julia was describing this moment, I watched her physically enact in the present what she was describing in the past. When she shared ‘*. . .I would look at my legs and think that I had too much fat on my legs, or my arms, above my shoulders. . .*’ Julia grabbed her legs, arms, and shoulders intensely in the present moment while sitting in a chair across from me. Julia was not aware of her doing so either. However, it was obvious to me the co-existence of the past and present entangled together and the reinforcement of how Julia holds memory and storied experiences through her physical body, and that time does not exist in a neat and linear timeline.

#### Venting Through Art

During one of the last conversational interviews, Julia explained how she recently utilized art as a tool to vent her emotions following a purging episode.

Julia: I do have one (drawing) that I made more recently. . . I was having a really bad night, and I had purged and I hadn’t done that in months so I was really stressed about it and I just wanted to get it out . . .the raw emotions that was going on there because it’s different from what I am usually drawing it’s not the same. I feel like that’s true for anyone who kind of makes artwork through their venting or something. . . it’s just like a completely different emotional feeling. . . You know there’s other ways to express how you’re feeling then just through words and that’s what I try, try to get out because like I bottle things up a lot. . . I also have a really hard time speaking about hard things and when I’m upset it’s hard to talk to people about it like I feel like I can’t open my mouth sometimes it feels like it’s really stuck in there (Julia grabbed her throat in the present, embodying the experience) you know like physically it feels like everything is stuck inside. So, I feel like drawing is a way to like put a pen in my hand and pen to the paper and it’s like all flowing out of me I guess without having to try to speak when it’s really hard. . .

While exploring this experience Julia came to a new insight and understanding about her eating disorder, and how art can sometimes tell her more about her emotions and feelings then words can.

Julia: It’s a very emotional illness, a lot of it comes from feelings that I don’t really understand . . . I’ve had people ask me before ‘where does this come from?’ ‘Are you doing this because you want to lose weight or your unhappy with the way you look?’. . . But I also have to try to think, why do I do this sometimes? why do I feel like I have to do this sometimes? And I just say I don’t know. I really, I don’t know, and it’s hard to figure those things out sometimes and drawing just helps me kind of figure out what am I feeling you know like where is this coming from. . . it’s just a different way of communicating, I guess, that I haven’t really thought of. I never really thought of that being a way to communicate with other people. . .

The use of art when working with individuals living with an eating disorder has been shown to promote self-expression, self-awareness, foster new perspectives, offer a sense of distraction, and produce a sense of pride (Griffin et al., 2021). Furthermore, art has been used as a method with this population to express challenging emotions, experiences, produce significant personal symbols, and tap into universal themes providing a different form of communication where language does not interfere (Griffin et al., 2021; Hinz, 2006). Current literature exploring art in relation to eating disorders remains limited, however, what does exist underscores its role in engaging aspects of experience that are difficult to articulate through language alone (Griffin et al., 2021; Hinz, 2006).

**Figure 1.**
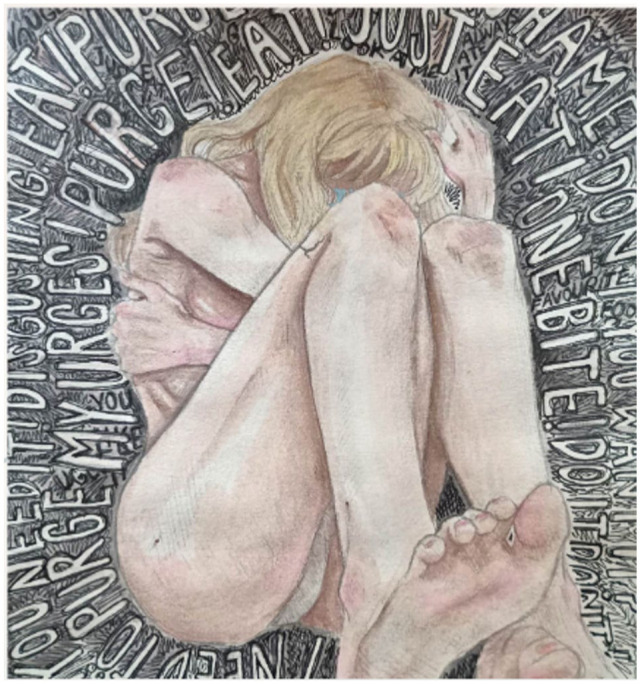
Julia, *Venting Through Art*, Mixed Media (graphite drawing), 2023, collection and copyright of the artist and author.

In the instance, Julia was able to explore and deepen both her own and my understanding of how art can be used to express and process the emotions and thoughts associated with an eating disorder. At the conclusion of the narrative inquiry study, she gifted me the artwork we had discussed, allowing it to serve as a tangible representation in future re-tellings of this experience. In addition to verbal consent provided by Julia to reproduce her artwork, written consent has been obtained to reproduce her artwork in future dissemination of scholarly activities. Figure 1 details this piece as a field artefact as a means for readers to gain access (Clandinin, 2013) to the complex, embodied, nature of an eating disorder illness so often difficult to represent in eating disorder literature. Furthermore, reflects a strength of narrative inquiry in its capacity to incorporate multiple forms of representation to engage with the layered and relational nature of lived experience.

## Implications and Contributions

Findings from this study build on existing eating disorder literature that challenges linear, biomedical notions of recovery and highlights the embodied dimensions of eating disorders. While much of this literature acknowledges ‘complexity’, it often does so conceptually rather than experientially. Narrative inquiry offers a distinctive way of engaging with ‘complexity’ because it attends to experience as storied, relational, and unfolding across time and place. By working with Julia and Emma’s narratives, this approach makes visible the negotiations, contradictions, and tensions that shape eating disorder conceptualization, care, and recovery in ways that are often flattened by methods seeking certainty or generalizability.

Narrative inquiry does not simply describe ‘complexity’, it creates space for it to be lived and understood through relational engagement, bringing forward layered experiences such as the co-existence of hope and struggle, and the embodied strategies used to navigate vulnerability. In doing so, narrative inquiry enacts an ontological openness that extends beyond current conceptual discussions, offering new possibilities for understanding and responding to eating disorders in both care and research.

This paper draws on narrative inquiry as a relational method that explores experience through story. With its commitment to relational research, narrative inquiry involves an in-depth exploration of complex phenomena that are poorly understood. Through the three-dimensional inquiry space, phenomena are better understood in the context of temporality, sociality, and place, allowing for a deeper understanding of how personal, social, and cultural narratives come together and shape the lives of individuals. Additionally, through its relational research method, narrative inquiry is well-positioned to meet the stated needs of persons living with an eating disorder regarding engaging in eating disorder research.

The purpose of narrative inquiry is not to make universal claims or generalizations but to create resonance through the rich description of lived experiences, stories that are told, relived, and retold, so readers can imagine their own uses and applications (Clandinin & Connelly, 2000; Clandinin & Murphy, 2009). Resonance reflects a deep sense of plausibility that allows readers to live vicariously through the stories and to gain a heightened awareness of the phenomenon under inquiry. Narrative inquiry researchers also acknowledge that there is never a single or final story; instead, each narrative opens into new possibilities for stories to be lived and told (Clandinin & Connelly, 2000; Connelly & Clandinin, 1990). Accordingly, the findings presented here speak for the ‘now’ of these co-constructed experiences, offering one meaningful way of understanding, while recognizing the evolving nature of lived experience.

Resonant threads explored within this paper included temporality and embodiment as key areas for future exploration in eating disorder conceptualization, care, and recovery. As evidenced in this study, participants described their eating disorder in a non-linear fashion, where at any given time, they can be experiencing their eating disorder based on past experiences, present experiences, or future hopes.

Within eating disorder literature, recovery is an elusive concept, with much debate about what defines eating disorder recovery (de Vos et al., 2017; Eli & Lavis, 2022; LaMarre & Rice, 2016). Traditionally, recovery has been conceptualized using a medical model with a primary focus on symptom management (de Vos et al., 2017; Kenny et al., 2019; Sternheim et al., 2021). However, the qualitative literature reviewed (de Vos et al., 2017; Kenny et al., 2019; LaMarre & Rice, 2016) highlights that defining recovery specifically around symptom management is not sufficient. In studies that included the perspectives of individuals in recovery found a distinction between clinically assessed eating disorder recovery and a personal, subjective sense of recovery (Bardone-Cone, 2012; Gillespie & Robinson, 2022). This growing body of scholarship is echoed in the experiences shared by co-participants in this study.

When pausing to consider what eating disorder recovery means to them, co-participants described that recovery was not defined by a specific weight restoration or the absence of an eating disorder. Instead, they envision a world of learning to live with the presence of an eating disorder but in a way that they would have autonomy over the characteristics of their eating disorder illness. Co-participants express that there must be a movement away from weight restoration-based recovery and attention placed on the psyche and individual experience of how they live with an eating disorder and why.

Both Julia and Emma’s experiences and narrative accounts support the budding exploration of the role of embodiment, how we perceive our body, its relationship to self, others, and environment, as a developing area of research looking to further understanding of eating disorder illness (Eli & Lavis, 2022; Fuchs, 2022; Osler, 2021). Within eating disorder literature, Clancy (2021) explored the concept of embodiment and how the affective forces (feelings, moods, attitudes) of trauma in anorexia and bulimia nervosa shape experiences and sensations of embodiment in maintaining an eating disorder. Clancy (2021) looked to add to the growing body of scholarship by moving away from what eating disorder behaviours do *to* a body and, rather, exploring what they do *for* a person. Clancy (2021) found that the majority of participants’ anorexia and/or bulimia were rooted in negative effects (fear, shame, disgust) and, as such, produced an intense vulnerability and sense of degradation that eating disorder behaviours work to stabilize and protect. Indeed, Pellegrini et al. (2021) express that eating disorders can be viewed as ‘embodied acts’ that help to cope with internal and external demands that are perceived as overwhelming.

It is important to note that there are several limitations to this study. The two participants in this study were both white women who use she/her pronouns, although one identified as a member of the 2SLGBTQIA+ community. This demographic homogeneity risks reinforcing dominant narratives that eating disorders primarily affect white females. While some similarities in the nature of the illness were observed, this lack of diversity is a limitation. While some similarities in illness experience were observed, the lack of diversity is a limitation. Efforts were made to situate participants’ stories within broader social, cultural, and institutional narratives; however, future research should include more diverse voices, including men, individuals from cultural or ethnic minority groups, and members of the 2SLGBTQIA+ community, populations historically underrepresented in eating disorder research. Additionally, although recruitment posters were placed across the province where this study took place, both participants lived in the same city. This may reflect challenges related to rurality, such as limited access, willingness to participate, and/or potential fear of identification. Future research could explore how rural contexts influence the experience of living with an eating disorder.

## Conclusion

Narrative inquiry is a relational and reflexive research methodology concerned with understanding and representing experience through story. Rather than having a pre-determined research question, the research puzzle evolves through the co-creation of knowledge between the researcher and participants. This paper detailed a narrative inquiry study that was conducted with two young adults living with an eating disorder, Julia and Emma. Field texts were analyzed using Clandinin & Connelly’s (2000) three-dimensional inquiry space and included conversational interviews and a reflexive journal. Field texts were analyzed for emerging narrative threads and shaped into interim research texts. Interim research texts transitioned into narrative accounts, which were reviewed by co-participants and shaped into a final research text that represented my Master of Nursing thesis.

Narrative threads of temporality and embodiment were highlighted as exemplars demonstrating how narrative inquiry as a research methodology can assist in deepening understanding of the complex nature of eating disorders. The use of a field artefact in the form of a visual art piece was presented as a means for readers to gain access to the experience of living with an eating disorder. Engaging in relational research not only responds to the stated needs of those living with an eating disorder, but brings forward a rich description of experience, which can illuminate new insights and understandings to the field of eating disorder research. As a result, can offer profound insights into the social, cultural, and institutional narratives that shape and uphold traditional conceptualization of eating disorders and open up how we think about and engage with the ‘complexity’ of eating disorder conceptualization, care and recovery.

## References

[bibr1-23333936261419040] American Psychiatric Association (APA). (2022). Diagnostic and statistical manual of mental disorders (5th ed., text rev.). American Psychiatric Association Publishing.

[bibr2-23333936261419040] Arnow K. D. Feldman T. Fichtel E. Lin I. H. Egan A. Lock J. Westerman M. Darcy A. M. (2017). A qualitative analysis of male eating disorder symptoms. Eating Disorders, 25(4), 297–309. 10.1080/10640266.2017.1308729 28394743

[bibr3-23333936261419040] Bardone-Cone A. M. (2012). Examining the match between assessed eating disorder recovery and subjective sense of recovery: Preliminary findings. European Eating Disorders Review, 20(3), 246–249. 10.1002/erv.112 21710559 PMC3184312

[bibr4-23333936261419040] Brelet L. Flaudias V. Désert M. Guillaume S. Llorca P. M. Boirie Y. (2021). Stigmatization toward people with anorexia nervosa, bulimia nervosa, and binge eating disorder: A scoping review. Nutrients, 13, 32834. 10.3390/nu13082834 PMC8400545 34444994

[bibr5-23333936261419040] Burnette C. B. Luzier J. L. Weisenmuller C. M. Boutté R. L. (2022). A systematic review of sociodemographic reporting and representation in eating disorder psychotherapy treatment trials in the United States. International Journal of Eating Disorders, 55(4), 423–454. 10.1002/eat.23699 35288967 PMC8988395

[bibr6-23333936261419040] Caine V. Estefan A. Clandinin D. J. (2013). A return to methodological commitment: Reflections on narrative inquiry. Scandinavian Journal of Educational Research, 57(6), 574–586. 10.1080/00313831.2013.798833

[bibr7-23333936261419040] Canadian Eating Disorders Alliance (CEDA). (2019). The Canadian eating disorders strategy: 2019–2029. https://nied.ca/wpcontent/uploads/2019/11/NIED_Strategy_Eng_CR_MedRes_SinglePages_REV.pdf

[bibr8-23333936261419040] Clancy E. (2021). ‘I feel fat when I feel fat’: Affective forces of trauma in anorexia and bulimia. Gender, Place & Culture, 29(3), 303–322. 10.1080/0966369X.2021.1873741

[bibr9-23333936261419040] Clandinin D. J. (2006). Narrative inquiry: A methodology for studying lived experience. Research Studies in Music Education, 27(1), 44–55. 10.1177/1321103X060270010301

[bibr10-23333936261419040] Clandinin D. J. (2013). Engaging in narrative inquiry. Left Coast Press.

[bibr11-23333936261419040] Clandinin D. J. Caine V. (2008). Narrative inquiry. In Given L. M. (Ed.), The SAGE encyclopedia of qualitative research methods (pp. 542–545). Sage. http://dx.doi.org/10.4135/9781412963909.n275

[bibr12-23333936261419040] Clandinin D. J. Caine V. Lessard S. Huber J. (2016). Engaging in narrative inquiries with children and youth. Taylor & Francis.

[bibr13-23333936261419040] Clandinin D. J. Cave M. T. Berendonk C. (2017). Narrative inquiry: A relational research methodology for medical education. Medical Education, 51(1), 89–96. 10.1111/medu.13136 27807868

[bibr14-23333936261419040] Clandinin D. J. Connelly F. M. (2000). Narrative inquiry: Experience and story in qualitative research (1st ed.). Jossey-Bass.

[bibr15-23333936261419040] Clandinin D. J. Murphy M. S. (2009). Comments on Coulter and Smith: Relational ontological commitments in narrative research. Educational Researcher, 38(8), 598–602. 10.3102/0013189X09353940

[bibr16-23333936261419040] Connelly F. M. Clandinin D. J. (1990). Stories of experience and narrative inquiry. Educational Researcher, 19(5), 2–14. 10.2307/1176100

[bibr17-23333936261419040] Corcoran R. Trainor G. Robinson B. (2021). The minority or the misunderstood? A young man’s journey with anorexia nervosa. Journal of Psychiatric & Mental Health Nursing, 28(5), 760–772. 10.1111/jpm.12784 34236744

[bibr18-23333936261419040] Corral-Liria I. Alonso-Maza M. González-Luis J. Fernández-Pascual S. Becerro-de-Bengoa-Vallejo R. Losa-Iglesias M. (2021). Holistic nursing care for people diagnosed with an eating disorder: A qualitative study based on patients and nursing professionals’ experience. Perspectives in Psychiatric Care, 58(2), 840–849. 10.1111/ppc.12858 34031892

[bibr19-23333936261419040] Couturier J. Isserlin L. Norris M. Spettigue W. Brouwers M. Kimber M. McVey G. Webb C. Findlay S. Bhatnagar N. Snelgrove N. Ritsma A. Preskow W. Miller C. Coelho J. Boachie A. Steinegger C. Loewen R. Loewen T. . . . Pilon D. (2020). Canadian practice guidelines for the treatment of children and adolescents with eating disorders. Journal of Eating Disorders, 8(4), 2–80. 10.1186/s40337-020-0277-8 32021688 PMC6995106

[bibr20-23333936261419040] Darcy A. M. Doyle A. C. Lock J. Peebles R. Doyle P. Le Grange D. (2012). The Eating Disorder Examination in adolescent males with anorexia nervosa: How does it compare to adolescent females? The International Journal of Eating Disorders, 45(1), 110–114. 10.1002/eat.20896 22170022 PMC3241004

[bibr21-23333936261419040] Davén J. Hellzen O. Häggström M. (2022). Encountering patients with anorexia nervosa—An emotional roller coaster. nurses’ lived experiences of encounters in psychiatric inpatient care. International Journal of Qualitative Studies on Health and Well-Being, 17(1), 2069651. 10.1080/17482631.2022.2069651 PMC9068011 35481811

[bibr22-23333936261419040] de Vos J. A. LaMarre A. Radstaak M. Bijkerk C. A. Bohlmeijer E. T. Westerhof G. J . (2017). Identifying fundamental criteria for eating disorder recovery: A systematic review and qualitative meta-analysis. Journal of Eating Disorders, 5(34), 1–14. 10.1186/s40337-017-0164-0 29118983 PMC5664841

[bibr23-23333936261419040] Dewey J. (1934). Art as experience. Berkley Publishing Group.

[bibr24-23333936261419040] Dewey J. (1938). Experience and education. Simon & Schuster.

[bibr25-23333936261419040] Dimitropoulos G. Freeman V. E. Muskat S. Domingo A. McCallum L. (2016). “You don’t have anorexia, you just want to look like a celebrity”: Perceived stigma in individuals with anorexia nervosa. Journal of Mental Health, 25(1), 47–54. 10.3109/09638237.2015.1101422 26651502

[bibr26-23333936261419040] Eli K. (2015). Binge eating as a meaningful experience in bulimia nervosa and anorexia nervosa: A qualitative analysis. Journal of Mental Health, 24(6), 363–368. 10.3109/09638237.2015.1019049 25992963

[bibr27-23333936261419040] Eli K. Lavis A. (2022). Material environments and the shaping of anorexic embodiment: Towards a materialist account of eating disorders. Culture, Medicine and Psychiatry, 46(2), 344–363. 10.1007/s11013-021-09715-8 33826076 PMC9034987

[bibr28-23333936261419040] Elwyn R. Adams M. Sharpe S. L. Silverstein S. LaMarre A. Downs J. Burnette C. B. (2024). Discordant conceptualisations of eating disorder recovery and their influence on the construct of terminality. Journal of Eating Disorders, 12(1), Article 70. 10.1186/s40337-024-01016-w PMC11145809 38831456

[bibr29-23333936261419040] Eshkevari E. Rieger E. Longo M. R. Haggard P. Treasure J. (2014). Persistent body image disturbance following recovery from eating disorders. The International Journal of Eating Disorders, 47(4), 400–409. 10.1002/eat.22219 24243423

[bibr30-23333936261419040] Finlay J. Dela Cruz A. (2023). Reflexivity and relational spaces: Experiences of conducting a narrative inquiry study with emerging adult women living with chronic pain. Global Qualitative Nursing Research, 10, 1–10. 10.1177/23333936231190619 PMC10413903 37576739

[bibr31-23333936261419040] Fuchs T. (2022). The disappearing body: Anorexia as a conflict of embodiment. Eating & Weight Disorders, 27(1), 109–117. 10.1007/s40519-021-01122-7 33666885 PMC8860785

[bibr32-23333936261419040] Gattario K. H. Frisén A. Teall T. L. Piran N. (2020). Embodiment: Cultural and gender differences and associations with life satisfaction. Body Image, 35, 1–10. 10.1016/j.bodyim.2020.07.005 32877841

[bibr33-23333936261419040] Gillespie C. W. Robinson E. G. (2022). Habits and attitudes about eating and self-weighing among adults who are recovered, recovering, or partially recovered from eating disorders: An open-ended survey study. Eating and Weight Disorders, 27(3), 1223–1228. 10.1007/s40519-021-01248-8 34185308

[bibr34-23333936261419040] Griffin C. Fenner P. Landorf K. B. Cotchett M. (2021). Effectiveness of art therapy for people with eating disorders: A mixed methods systematic review. The Arts in Psychotherapy, 76, Article 101859. 10.1016/j.aip.2021.101859

[bibr35-23333936261419040] Griffiths S. Yager Z. (2019). Gender, embodiment, and eating disorders. The Journal of Adolescent Health, 64(4), 425–426. 10.1016/j.jadohealth.2019.01.016 30904090

[bibr36-23333936261419040] Gustafsson S. A. Stenström K. Olofsson H. Pettersson A. Wilbe Ramsay K. (2021). Experiences of eating disorders from the perspectives of patients, family members, and health care professionals: A meta-review of qualitative evidence syntheses. Journal of Eating Disorders, 9(1), Article 150. 10.1186/s40337-021-00507-4 PMC8642844 34863276

[bibr37-23333936261419040] Hartman-Munick S. M. Silverstein S. Guss C. E. Lopez E. Calzo J. P. Gordon A. R. (2021). Eating disorder screening and treatment experiences in transgender and gender diverse young adults. Eating Behaviors, 41, Article 101517. 10.1016/j.eatbeh.2021.101517 PMC9645530 33962139

[bibr38-23333936261419040] Hinz L. D. (2006). Drawing from within: Using art to treat eating disorders. Jessica Kingsley Publishers.

[bibr39-23333936261419040] Joy L. Jones A. White C. (2021). Safe, seen, & supported: Navigating eating disorders recovery in the 2SLGBTQ+ community. Eating Disorders Nova Scotia. https://ln5.sync.com/dl/8929879f0/pi5ft5xh-ccm23rvg-8skusnfb-jikfv5dc 10.3148/cjdpr-2022-033 36413404

[bibr40-23333936261419040] Jennings K. M. (2017). The Roy Adaptation Model: A theoretical framework for nurses providing care to individuals with anorexia nervosa. Advances in Nursing Science, 40(4), 370–383. 10.1097/ans.0000000000000175 28825933 PMC5664223

[bibr41-23333936261419040] Katznelson H. Daniel S. I. F. Poulsen S. Lunn S. Buhl-Nielsen B. Sjögren J. M. (2021). Disturbances in the experiences of embodiment related to attachment, mentalization and self-objectification in anorexia nervosa. Journal of Eating Disorders, 9(1), Article 137. 10.1186/s40337-021-00463-z PMC8542305 34688309

[bibr42-23333936261419040] Kenny T. Boyle S. Lewis S. (2019). #Recovery: Understanding recovery from the lens of recovery-focused blogs posted by individuals with lived experience. International Journal of Eating Disorders, 53, 1234–1243. 10.1002/eat.23221 31886573

[bibr43-23333936261419040] King S. J. Turner D. S. (2000). Caring for adolescent females with anorexia nervosa: Registered nurses’ perspective. Journal of Advanced Nursing, 32(1), 139–147. 10.1046/j.1365-2648.2000.01451.x 10886445

[bibr44-23333936261419040] Klein D. A. Sylvester J. Schvey N. A. (2021). Eating disorders in primary care: Diagnosis and management. American Academy of Family Physicians, 103(1), 22–32. https://www.aafp.org/afp/2021/0101/p22.html 33382560

[bibr45-23333936261419040] Koruth N. Nevison C. Schwannauer M. (2012). A grounded theory exploration of the onset of anorexia in adolescence. European Eating Disorders Review, 20(4), 257–264. 10.1002/erv.1135 21710572

[bibr46-23333936261419040] LaMarre A. Rice C. (2016). Normal eating is counter-cultural: Embodied experiences of eating disorder recovery. Journal of Community & Applied Social Psychology, 26(2), 136–149. 10.1002/casp.2240

[bibr47-23333936261419040] Lavis A. (2018). Not eating or tasting other ways to live: A qualitative analysis of “living through” and desiring to maintain anorexia. Transcultural Psychiatry, 55(4), 454–474. 10.1177/1363461518785796 30056795

[bibr48-23333936261419040] Law J. Mol A. (2002). In A. Mol (Ed.), Complexities: An introduction. Complexities: Social Studies of Knowledge Practices (pp. 1–22). Duke University Press. 10.2307/j.ctv113144n

[bibr49-23333936261419040] LeBlanc H. (2014). Eating disorders among girls and women in Canada. Report of the Standing Committee on the Status of Women. https://www.ourcommons.ca/DocumentViewer/en/41-2/FEWO/report-4

[bibr50-23333936261419040] Lester R. J. (2021). Famished: Eating disorders and failed care in America. University of California Press.

[bibr51-23333936261419040] Malecki J. Rhodes P. Ussher J. (2018). Childhood trauma and anorexia nervosa: From body image to embodiment. Health Care for Women International, 39(8), 936–951. 10.1080/07399332.2018.1492268 30152723

[bibr52-23333936261419040] Malson H. Tischner I. Herzig H. Kitney D. Phillipps C. Norweg S. Moon J. Holmes S. Wild K. Oldham-Cooper R. (2022). Key stakeholder perspectives on primary care for young people with an eating disorder: A qualitative study. Journal of Community & Applied Social Psychology, 32(2), 288–301. 10.1002/casp.2575

[bibr53-23333936261419040] Medway M. Rhodes P. Dawson L. Miskovic-Wheatley J. Wallis A. Madden S. (2019). Adolescent development in family-based treatment for anorexia nervosa: Patients’ and parents’ narratives. Clinical Child Psychology and Psychiatry, 24(1), 129–143. 10.1177/1359104518792293 30080102

[bibr54-23333936261419040] Obeid N. McVey G. Seale E. Preskow W. Norris M. L. (2020). Cocreating research priorities for anorexia nervosa: The Canadian eating disorder priority setting partnership. International Journal of Eating Disorders, 53, 662–672. 10.1002/eat.23234 32011022

[bibr55-23333936261419040] Okri B. (1997). A way of being free. Phoenix House.

[bibr56-23333936261419040] Ortiz S. Espel-Huynh H. M. Felnois C. Scharff A. (2019). Qualitative perceptions of and preferences for the research process among patients with eating disorders. International Journal of Eating Disorders, 53, 41–51. 10.1002/eat.23176 31617609

[bibr57-23333936261419040] Osler L. (2021). Controlling the noise: A phenomenological account of anorexia nervosa and the threatening body. Philosophy, Psychiatry, & Psychology, 28(1), 41–58. 10.1353/ppp.2021.0008

[bibr58-23333936261419040] Pellegrini R. A. Finzi S. Veglia F. Di Fini G. (2021). Narrative and bodily identity in eating disorders: Toward an integrated theoretical-clinical approach. Frontiers in Psychology, 12, Article 785004. 10.3389/fpsyg.2021.785004 PMC8714898 34975677

[bibr59-23333936261419040] Rossi E. Castellini G. Cassioli E. Sensi C. Mancini M. Stanghellini G. Ricca V. (2021). The role of embodiment in the treatment of patients with anorexia and bulimia nervosa: A 2-year follow-up study proposing an integration between enhanced cognitive behavioural therapy and a phenomenological model of eating disorders. Eating and Weight Disorders, 26(8), 2513–2522. 10.1007/s40519-021-01118-3 33534077 PMC7856332

[bibr60-23333936261419040] Ryan V. Malson H. Clarke S. Anderson G. Kohn M. (2006). Discursive constructions of ‘eating disorders nursing’: An analysis of nurses’ accounts of nursing eating disorder patients. European Eating Disorders Review, 14(2), 125–135. 10.1002/erv.666

[bibr61-23333936261419040] Sternheim L. C. Wickham M. I. Danner U. N. Maddox T. W. Filoteo V. J. Shott M. E. Frank G. K. W. (2021). Understanding implicit and explicit learning in adolescents with and without anorexia nervosa. Journal of Eating Disorders, 9, Article 77. 10.1186/s40337-021-00431-7 PMC8243584 34187577

[bibr62-23333936261419040] Urban B. Smith E. K. Adams M. Sharpe S. L. Silverstein S. (2024). Guidelines for research with transgender, gender diverse, and intersex individuals with eating disorders: Recommendations from trans and intersex researchers. Eating Disorders, 32(4), 341–352. 10.1080/10640266.2024.2306436 38334066

[bibr63-23333936261419040] van Doornik S. F. W. Ostafin B. D. Jonker N. C. Glashouwer K. A. de Jong P. J . (2021). Low satisfaction with normative life domains in adolescents with anorexia nervosa. Clinical Psychology & Psychotherapy, 28(5), 1266–1274. 10.1002/cpp.2574 33608934 PMC8596741

[bibr64-23333936261419040] Wu W. Chen S. (2021). Nurses’ perceptions of and experiences in conflict situations when caring for adolescents with anorexia nervosa: A qualitative study. International Journal of Mental Health Nursing, 30(Suppl. 1), 1386–1394. 10.1111/inm.12886 34047043

